# Adverse cardiovascular magnetic resonance phenotypes are associated with greater likelihood of incident coronavirus disease 2019: findings from the UK Biobank

**DOI:** 10.1007/s40520-021-01808-z

**Published:** 2021-03-08

**Authors:** Zahra Raisi-Estabragh, Celeste McCracken, Jackie Cooper, Kenneth Fung, José M. Paiva, Mohammed Y. Khanji, Elisa Rauseo, Luca Biasiolli, Betty Raman, Stefan K. Piechnik, Stefan Neubauer, Patricia B. Munroe, Nicholas C. Harvey, Steffen E. Petersen

**Affiliations:** 1 William Harvey Research Institute, NIHR Barts Biomedical Research Centre, Queen Mary University of London, Charterhouse Square, London, EC1M 6BQ UK; 2 Barts Heart Centre, St Bartholomew’s Hospital, Barts Health NHS Trust, London, EC1A 7BE UK; 3 National Institute for Health Research Oxford Biomedical Research Centre, Division of Cardiovascular Medicine, Radcliffe Department of Medicine, University of Oxford, Oxford, UK; 4 MRC Lifecourse Epidemiology Unit, University of Southampton, Southampton, UK; 5 NIHR Southampton Biomedical Research Centre, University of Southampton and University Hospital Southampton NHS Foundation Trust, Southampton, UK

**Keywords:** Cardiovascular magnetic resonance, Coronavirus disease 2019 (COVID-19), Severe acute respiratory syndrome coronavirus 2 (SARS-CoV-2)

## Abstract

**Background:**

Coronavirus disease 2019 (COVID-19) disproportionately affects older people. Observational studies suggest indolent cardiovascular involvement after recovery from acute COVID-19. However, these findings may reflect pre-existing cardiac phenotypes.

**Aims:**

We tested the association of baseline cardiovascular magnetic resonance (CMR) phenotypes with incident COVID-19.

**Methods:**

We studied UK Biobank participants with CMR imaging and COVID-19 testing. We considered left and right ventricular (LV, RV) volumes, ejection fractions, and stroke volumes, LV mass, LV strain, native T1, aortic distensibility, and arterial stiffness index. COVID-19 test results were obtained from Public Health England. Co-morbidities were ascertained from self-report and hospital episode statistics (HES). Critical care admission and death were from HES and death register records. We investigated the association of each cardiovascular measure with COVID-19 test result in multivariable logistic regression models adjusting for age, sex, ethnicity, deprivation, body mass index, smoking, diabetes, hypertension, high cholesterol, and prior myocardial infarction.

**Results:**

We studied 310 participants (*n* = 70 positive). Median age was 63.8 [57.5, 72.1] years; 51.0% (*n* = 158) were male. 78.7% (*n* = 244) were tested in hospital, 3.5% (*n* = 11) required critical care admission, and 6.1% (*n* = 19) died. In fully adjusted models, smaller LV/RV end-diastolic volumes, smaller LV stroke volume, and poorer global longitudinal strain were associated with significantly higher odds of COVID-19 positivity.

**Discussion:**

We demonstrate association of pre-existing adverse CMR phenotypes with greater odds of COVID-19 positivity independent of classical cardiovascular risk factors.

**Conclusions:**

Observational reports of cardiovascular involvement after COVID-19 may, at least partly, reflect pre-existing cardiac status rather than COVID-19 induced alterations.

**Supplementary Information:**

The online version contains supplementary material available at 10.1007/s40520-021-01808-z.

## Introduction

Coronavirus disease 2019 (COVID-19), which disproportionately affects older people, is increasingly recognised as a multi-system disease [1]. A spectrum of cardiovascular manifestations has been reported in the context of acute infection [2–8]. The high inflammatory burden of acute COVID-19 is postulated to lead to vascular inflammation, myocarditis, and cardiac arrhythmias [9–12]. The accompanying hypercoagulable state has been linked to higher rates of thromboembolic events manifesting as myocardial and cerebral infarctions [13].

Interestingly, acute myocardial injury, occurring in the context of COVID-19, has been linked to greater mortality independent of factors such as acute respiratory distress syndrome [5, 6]. Reports of primary cardiac presentations of COVID-19 in the absence of pulmonary involvement suggest potential cardio-specific actions of severe acute respiratory syndrome coronavirus 2 (SARS-CoV-2) [14–16]. Indeed, in vitro studies have demonstrated that human cardiomyocytes are permissive for SARS-CoV-2 infection and that the virus induces a cytotoxic response in these cells [17]. Similarly, autopsy studies have demonstrated direct cardiotoxic effects of SARS-CoV-2 [18].

In addition to cardiac involvement linked with acute COVID-19, an emerging subset of individuals report a protracted disease course with a range of potential cardiac symptoms persisting for many weeks after the acute illness [19–21]. Limited observational studies have suggested sustained cardiac involvement, based on cardiovascular magnetic resonance (CMR) assessment, after recovery from acute COVID-19 [22–25]. However, the lack of baseline CMR data severely limits any causal interpretation of such findings. We, therefore, investigated whether pre-existing CMR phenotypes were associated with risk of subsequent positive COVID-19 status in the UK Biobank (UKB).

## Methods

### Setting

The UK Biobank (UKB) is a very large cohort study incorporating data from over half a million participants recruited between 2006 and 2010, designed primarily for study of illnesses of middle and older age. Individuals aged 40–69 years were identified through National Health Service (NHS) registers and recruited via postal invites. There was detailed baseline characterisation of participants including socio-demographics, lifestyle, health status, and a series of physical measures [26]. Individuals who were unable to consent, or to complete baseline assessment due to illness or discomfort, were not recruited. The UKB protocol is publicly available [27]. Linkages with hospital episode statistics (HES) and death registers allow prospective tracking of health outcomes for all participants, documented according to international classification of disease (ICD) codes. The UKB has also produced algorithmically defined outcomes for key illnesses, such as acute myocardial infarction (AMI), which integrate data from several sources [28]. The UKB Imaging Study, which includes CMR imaging, aims to scan a random subset of 100,000 participants (> 48,000 completed, January 2021) [29]. Linkage with Public Health England permits tracking of COVID-19 test results for UKB participants [30, 31].

### COVID-19 tests

We included all UKB participants with CMR imaging and COVID-19 testing between 16/03/2020 and 22/08/2020. Testing was based on real-time polymerase chain reaction (RT-PCR) assay antigen testing. For most participants, the sample tested was from combined nose and throat swabs; for patients in critical care lower respiratory samples may have been used. We considered samples labelled as inpatient to be from a hospital setting. Critical care admissions and deaths were defined based on HES and death register data.

### CMR image acquisition and analysis

CMR imaging was performed in dedicated UKB imaging centres using uniform staff training, equipment, and according to a previously published protocol [32]. Image analysis was performed blind to all participant details using Circle Cardiovascular Imaging 42 post-processing software (Version 5.11, Circle Cardiovascular Imaging Inc., Calgary, Canada). The automated contour detection tool was used for analysis of ventricular volumes, LV mass, and LV strain (tissue tracking). There was visual quality control (QC) of all studies. Those with suboptimal contouring were manually corrected by a single reader. Native T1 was calculated from a manually drawn septal contour on a mid-ventricular short axis slice, excluding cases with poor quality maps or excess septal motion. Aortic distensibility (AoD) was calculated by dividing aortic strain (change in aortic cross-sectional area in diastole to systole) by central pulse pressure (CPP, mmHg) [33]. Aortic areas were derived from transverse cine images of the aorta using an automated tool previously developed and validated in the UKB [34]. The pipeline has inbuilt QC, which assigns a probability to correct detection of aortic areas. We limited analysis to measurements with correct detection probability > 0.75. Aortic contours were used to calculate aortic strain at both the ascending and descending aorta, which was divided by CPP taken from Vicorder® readings at time of imaging to derive AoD at both sites. Thus, the following CMR metrics were considered: LV/RV volumes in end-diastole and end-systole, LV/RV stroke volume, LV/RV ejection fraction, LV mass, mid-ventricular radial strain, mid-ventricular circumferential strain, global longitudinal strain (GLS), torsion, septal native T1, AoD at the ascending and descending aorta.

### Arterial stiffness index

As an additional measure of arterial health in a larger sample, we considered arterial stiffness index (ASI) derived from finger plethysmography [35]. ASI was measured at the baseline UKB visit using the PulseTrace PCA2 (CareFusion, USA) device according to a pre-defined protocol [36]. Outliers were removed from the ASI variable using the Tukey outlier removal method (1.5 $\times$ interquartile range (IQR) rule), as previously published using this dataset [37].

### Statistical analysis

Statistical analysis was performed using R Version 3.6.2 and RStudio Version 1.2.5019 [38, 39]. Summary descriptive statistics are presented as mean (standard deviation) or median [IQR] depending on distribution. We estimated the association of each cardiovascular phenotype measure with COVID-19 test result (positive vs negative) in logistic regression models with adjustment for age, sex, ethnicity, deprivation, body mass index (BMI), smoking, diabetes, hypertension, high cholesterol, and prior AMI. We present odds ratios with corresponding 95% confidence intervals (CIs) and *p *values per unit increase for each cardiac measure. We performed sensitivity analysis limiting to tests performed in a hospital setting (positive *n* = 50). In addition, owing to the relatively small number of positive cases, we re-ran the models using Firth’s penalised logistic regression [40], which produced near-identical results. Within the sample with COVID-19 testing and ASI measurement, we tested association of ASI with death and critical care admission separately in the test positive and negative cohorts with multivariable adjustment as before. The results were near identical in sensitivity analysis with positive cases defined based on RT-PCR and clinical diagnosis of COVID-19 as per HES records (ICD10 code H07.2).

### Ascertainment of covariates

We took sex as recorded at the baseline UKB visit and age at time of COVID-19 testing. We classified ethnicity into a binary variable of White and BAME (Black, Asian, and minority ethnic) ethnicities. UKB records the Townsend score as a measure of material deprivation, with negative scores indicating less deprivation than national averages [41]. BMI was calculated from height and weight recorded at baseline. Smoking status was based on self-report. Hypertension, diabetes, and hypercholesterolaemia were defined through cross-checking across self-report and HES data. A list of International Classification of Disease (ICD) codes used is presented in Supplementary Table 1. Prior AMI was obtained from UKB algorithmically defined health outcomes.

### Ethics

This study was covered by the ethical approval for UKB studies from the NHS National Research Ethics Service on 17th June 2011 (Ref 11/NW/0382) and extended 10th May 2016 (Ref 16/NW/0274). Access to UKB was granted through access application 2964. All procedures were performed in concordance with the Declaration of Helsinki.

## Results

### Characteristics of study participants

**Fig. 1:**
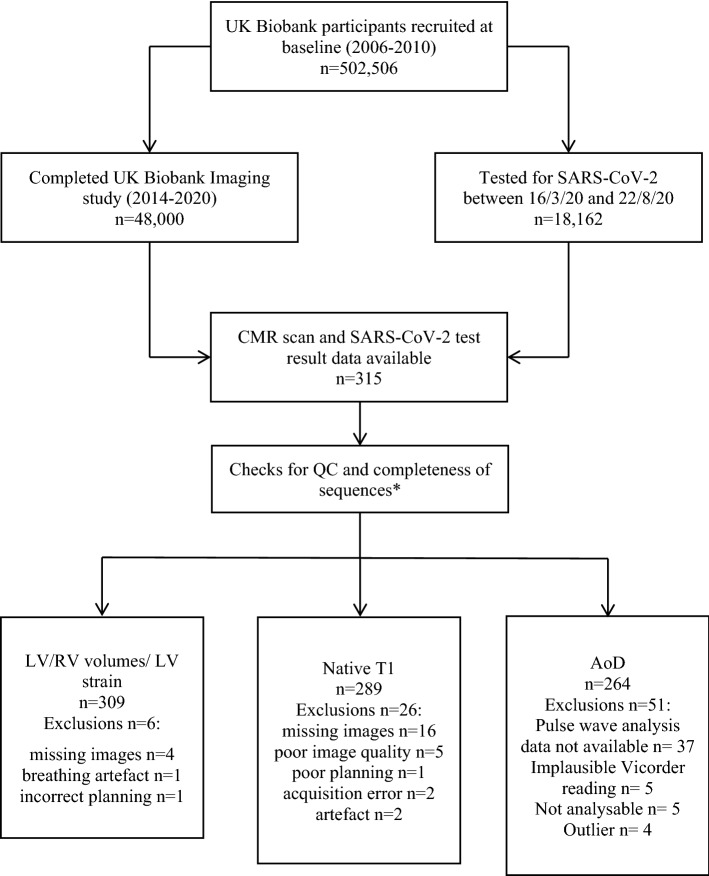
Approach to selection of participants for inclusion in the analysis. There were 310 participants with at least one analysable CMR measure, this included one participant with analysable native T1, but not volumetric images. *AoD* aortic distensibility; *CMR* cardiovascular magnetic resonance; *LV* left ventricle; *RV* right ventricle; *SARS-CoV-2* severe acute respiratory syndrome coronavirus 2

There were 315 UKB participants with CMR imaging and COVID-19 testing during the defined study period. The analysis sample comprised 310 participants (*n* = 70 positive) with at least one analysable CMR measure (Fig. 1). Of these, 78.7% (*n* = 244) were tested in hospital, 3.5% (*n* = 11) required critical care admission, and 6.1% (*n* = 19) died. Median age was 63.8 [57.5, 72.1] years; 51.0% (*n* = 158) were male (Table 1). Average interval between CMR and COVID-19 testing was 3.0 years. The rates of smoking, diabetes, hypertension, high cholesterol, and previous AMI were 45.2%, 9.4%, 40.6%, 29.4%, and 3.5%, respectively (Table 1).

**Table 1:** Characteristics of study participants

	Whole sample *n* = 310	COVID-19 negatives *n* = 240	COVID-19 positives *n* = 70	*p* value [test]
Age	63.8 [57.5, 72.1]	65.1 [58.2, 72.1]	61.4 [55.5, 72.4]	0.154 [b]
Sex (male)	158 (51.0%)	117 (48.8%)	41 (58.6%)	0.190 [c]
White	295 (95.2%)	232 (96.7%)	63 (90.0%)	0.049 [c]
BAME	15 (4.8%)	8 (3.3%)	7 (10.0%)	
Asian	8 (2.6%)	5 (2.1%)	3 (4.3%)	0.046 [d]
Black	2 (0.6%)		2 (2.9%)	
Mixed	3 (1.0%)	2 (0.8%)	1 (1.4%)	
Other	2 (0.6%)	1 (0.4%)	1 (1.4%)	
Townsend deprivation score	−2.5 [−3.8, 0.2]	−2.5 [−3.8, 0.0]	−2.4 [−4.1, 0.4]	0.960 [b]
Smoking (current/previous)	140 (45.2%)	108 (45.0%)	32 (45.7%)	1.000 [c]
BMI	27.4 (± 4.9)	27.3 (± 4.7)	27.6 (± 5.6)	0.629 [a]
Diabetes	29 (9.4%)	24 (10.0%)	5 (7.1%)	0.641 [d]
Hypertension	126 (40.6%)	101 (42.1%)	25 (35.7%)	0.414 [c]
High cholesterol	91 (29.4%)	70 (29.2%)	21 (30.0%)	1.000 [c]
Prior myocardial infarction	11 (3.5%)	10 (4.2%)	1 (1.4%)	0.466 [d]
Tested in hospital	244 (78.7%)	194 (80.8%)	50 (71.4%)	0.127 [c]
Critical care admission	11 (3.5%)	7 (2.9%)	4 (5.7%)	0.276 [d]
Death	19 (6.1%)	11 (4.6%)	8 (11.4%)	0.069 [c]

[a] Welch two sample *t* test (numeric data with unequal variances); [b] Wilcoxon rank sum test with continuity correction (numeric skewed); [c] Two-sample test for equality of proportions with continuity correction (where minimum count > 5); [d] Fisher's exact test for count data (where minimum count $\le$ 5)

*BAME* black, asian, and minority ethnic; *BMI* body mass index; *COVID-19* coronavirus disease 2019

Those with a positive test included greater proportions of men and BAME individuals than those testing negative (Table 1). There were greater number of critical care admissions (5.7% vs 2.9%) and deaths (11.4% vs 4.6%) in the COVID-19 positive group, but this was not statistically significant. Within the COVID-19 positives, those who required critical care admission had higher average rates of all cardiometabolic morbidities, higher BMI, more deprivation, and comprised a greater proportion of men (Supplementary Table 2); those who died also had poorer cardiometabolic profiles and were older than those who survived. Similar, but less pronounced, differences were observed in the COVID-19 negative group (Supplementary Table 2).

ASI was available for 167,423 participants at baseline, of those 6160 had COVID-19 testing within the study period. After outlier removal (*n* = 94), 6066 participants had analysable ASI, of whom 667 tested positive and 5399 tested negative. The baseline characteristics for this sample are summarised in Supplementary Table 3.

### Baseline cardiovascular phenotypes

**Fig. 2:**
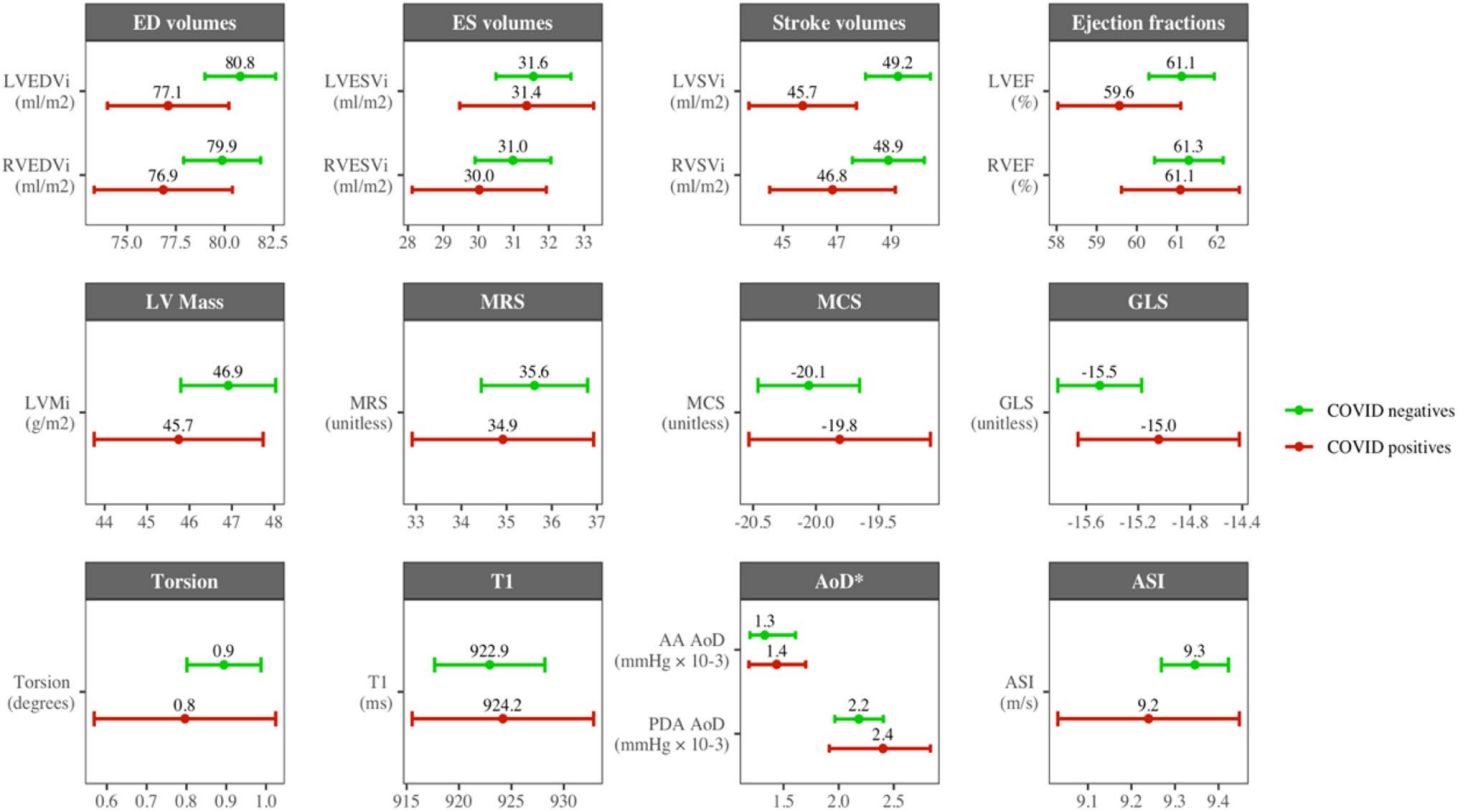
Summary of cardiovascular phenotype measures in the COVID-19 positive and negative groups. *AA AoD* aortic distensibility at the ascending aorta; *ASI* arterial stiffness index; *CMR* cardiovascular magnetic resonance; *COVID-19* coronavirus disease 2019; *ED* end-diastole; *ES* end-systole; *GLS* global longitudinal strain; *MCS* circumferential strain at the mid short axis level; *MRS* radial strain at the mid short axis level; *LV* left ventricle; *PDA AoD* aortic distensibility at the proximal descending aorta. Intervals for AoD show the 95% confidence interval for the median, all others are 95% confidence interval for the mean

Compared to those testing negative, the COVID-19 positive group had, on average, smaller LV end-diastolic volumes, smaller stroke volume, lower ejection fraction, and lower LV mass (Table 2, Fig. 2). There was a similar pattern in the RV measures with smaller volumes in end-diastole and end-systole and lower RV stroke volume. COVID-19 positives had, on average, poorer myocardial deformation by all strain measures (circumferential, global longitudinal, radial, torsion). They also had, compared to the COVID-19 negatives, slightly higher average native T1 and higher arterial compliance (higher AoD, lower ASI); however, there was near complete overlap of distributions for these variables (Table 2, Fig. 2, Supplementary Table 3).

**Table 2:** Summary of Cardiovascular magnetic resonance measures

	Whole sample *n* = 310	COVID-19 negatives *n* = 240	COVID-19 positives *n* = 70	*p *value [test]
LVEDVi (ml/m2)	80.0 (± 14.2)	80.8 (± 14.3)	77.1 (± 13.2)	0.046 [a]
LVESVi (ml/m2)	31.5 (± 8.3)	31.6 (± 8.4)	31.4 (± 8.1)	0.863 [a]
LVSVi (ml/m2)	48.5 (± 9.3)	49.2 (± 9.4)	45.7 (± 8.4)	0.004 [a]
LVEF (%)	60.8 (± 6.5)	61.1 (± 6.4)	59.6 (± 6.5)	0.083 [a]
LVMi (g/m2)	46.7 (± 8.8)	46.9 (± 8.8)	45.7 (± 8.4)	0.318 [a]
RVEDVi (ml/m2)	79.2 (± 15.5)	79.9 (± 15.6)	76.9 (± 15.0)	0.148 [a]
RVESVi (ml/m2)	30.8 (± 8.4)	31.0 (± 8.5)	30.0 (± 8.1)	0.393 [a]
RVSVi (ml/m2)	48.4 (± 10.4)	48.9 (± 10.5)	46.8 (± 9.8)	0.133 [a]
RVEF (%)	61.3 (± 6.6)	61.3 (± 6.8)	61.1 (± 6.2)	0.808 [a]
Native T1 (ms)	923.2 (± 39.1)	922.9 (± 40.1)	924.2 (± 35.9)	0.808 [a]
MRS (%)	35.5 (± 9.1)	35.6 (± 9.3)	34.9 (± 8.5)	0.558 [a]
MCS (%)	−20.0 (± 3.2)	−20.1 (± 3.2)	−19.8 (± 3.1)	0.562 [a]
GLS (%)	−15.4 (± 2.6)	−15.5 (± 2.5)	−15.0 (± 2.6)	0.206 [a]
Torsion (degrees)	0.9 (± 0.8)	0.9 (± 0.7)	0.8 (± 1.0)	0.437 [a]
AA AoD (× 10^−3^ mmHg^−1^)	1.4 [0.8, 2.3]	1.3 [0.8, 2.3]	1.4 [0.9, 2.3]	0.665 [b]
PDA AoD (× 10^−3^ mmHg^−1^)	2.2 [1.7, 3.1]	2.2 [1.7, 3.1]	2.4 [1.6, 3.2]	0.511 [b]

[a] Welch two sample *t* test (numeric data with unequal variances); [b] Wilcoxon rank sum test with continuity correction (numeric skewed)

*AA* ascending aorta; *AoD* aortic distensibility; *COVID-19* coronavirus disease 2019; *I* denotes indexation to body surface area; *LVEDV* left ventricular endo-diastolic volume; *LVEF* left ventricular ejection fraction; *LVESV* left ventricular endo-systolic volume; *LVSV* left ventricular stroke volume; *GLS*: global longitudinal strain; *MCS* circumferential strain at the mid short axis level; *MRS*: radial strain at the mid short axis level; *PDA* proximal descending aorta; *RVEDV* right ventricular endo-diastolic volume; *RVEF* right ventricular ejection fraction; *RVESV* right ventricular end-systolic volume; *RVSV* right ventricular stroke volume

COVID-19 positive individuals who died had significantly lower LV stroke volume, poorer GLS, and lower arterial compliance (lower AoD, higher ASI) compared to those who survived (Supplementary Table 4). Those who died in the COVID-19 negative group did not have statistically different LV measures compared to those who survived, but they did have higher ASI. It is plausible that the associations with adverse CMR phenotypes may be modified with severity of COVID-19. However, we were underpowered to definitively test associations with more granular outcomes, such as death or critical care admission.

### Association of cardiovascular phenotypes with COVID-19 status

**Fig. 3:**
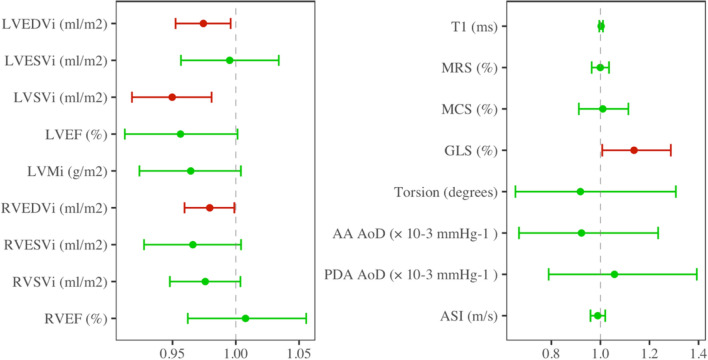
Odds ratios from fully adjusted multivariable logistic regression models demonstrating association of cardiovascular phenotype measures with COVID-19 status. Association of each cardiovascular measure with COVID-19 test result (positive vs negative) in multivariable logistic regression models adjusting for age, sex, ethnicity, deprivation, body mass index, smoking, diabetes, hypertension, high cholesterol, and prior myocardial infarction. Results are from individual models and expressed as odds ratio and 95% confidence interval (CI) corresponding to each cardiovascular measure. Green: 95% CI includes one. Red: 95% CI does not include one

In fully adjusted models, smaller LV and RV volumes in end-diastole, lower LV stroke volume, and poorer myocardial deformation by GLS (higher values) were associated with significantly higher odds of a positive COVID-19 test (Table 3, Fig. 3). Associations with other strain measures, torsion, and native T1 were not statistically significant. There were no significant associations between measures of arterial stiffness (AoD, ASI) and COVID-19 status.

**Table 3:** Odds ratios from logistic regression models demonstrating association of cardiovascular phenotype measures with COVID-19 status

	Univariate	Age and sex adjusted	Fully adjusted
LVEDVi (ml/m2)	0.98 [0.96, 1.00]	0.97* [0.95, 0.99]	0.97* [0.95, 1.00]
	0.057	8.50 × 10^−3^	0.022
LVESVi (ml/m2)	1.00 [0.96, 1.03]	0.99 [0.95, 1.02]	1.00 [0.96, 1.03]
	0.865	0.416	0.806
LVSVi (ml/m2)	0.96* [0.93, 0.99]	0.95* [0.92, 0.98]	0.95* [0.92, 0.98]
	6.4 × 10^−3^	1.4 × 10^−3^	2.2 × 10^−3^
LVEF (%)	0.96 [0.93, 1.00]	0.97 [0.93, 1.01]	0.96 [0.91, 1.00]
	0.081	0.127	0.059
LVMi (g/m2)	0.98 [0.95, 1.02]	0.96* [0.92, 1.00]	0.96 [0.92, 1.00]
	0.328	0.036	0.087
RVEDVi (ml/m2)	0.99 [0.97, 1.00]	0.98* [0.96, 1.00]	0.98* [0.96, 1.00]
	0.155	0.023	0.042
RVESVi (ml/m2)	0.99 [0.95, 1.02]	0.96* [0.92, 1.00]	0.97 [0.93, 1.00]
	0.404	0.050	0.087
RVSVi (ml/m2)	0.98 [0.95, 1.01]	0.97 [0.95, 1.00]	0.98 [0.95, 1.00]
	0.146	0.065	0.093
RVEF (%)	1.00 [0.96, 1.04]	1.01 [0.97, 1.06]	1.01 [0.96, 1.06]
	0.816	0.682	0.746
Native T1 (ms)	1.00 [0.99, 1.01]	1.00 [1.00, 1.01]	1.00 [1.00, 1.01]
	0.818	0.536	0.483
MRS (%)	0.99 [0.96, 1.02]	1.00 [0.97, 1.04]	1.00 [0.96, 1.03]
	0.575	0.828	0.972
MCS (%)	1.02 [0.94, 1.11]	0.99 [0.90, 1.09]	1.01 [0.91, 1.11]
	0.569	0.914	0.851
GLS (%)	1.07 [0.96, 1.19]	1.10 [0.98, 1.24]	1.14* [1.01, 1.29]
	0.197	0.113	0.039
Torsion (degrees)	0.86 [0.62, 1.20]	0.88 [0.63, 1.24]	0.92 [0.65, 1.31]
	0.366	0.443	0.628
AA AoD (× 10^−3^ mmHg^−1^)	1.06 [0.84, 1.33]	0.93 [0.68, 1.23]	0.92 [0.67, 1.24]
	0.602	0.625	0.606
PDA AoD (× 10^−3^ mmHg^−1^)	1.12 [0.89, 1.41]	1.04 [0.79, 1.35]	1.06 [0.79, 1.39]
	0.372	0.863	0.812
ASI (m/s)	0.99 [0.96, 1.02]	0.99 [0.96, 1.02]	0.99 [0.96, 1.02]
	0.367	0.549	0.481

Modelling is with sample tested for COVID-19 with analysable CMR data. Model outcome is set as COVID-19 test result (positive vs negative). Fully adjusted model includes adjustment for age, sex, ethnicity, deprivation, body mass index, smoking, diabetes, hypertension, high cholesterol, and prior myocardial infarction. Results are odds ratio [95% confidence interval] and *p* value, each belonging to a separate logistic regression model with covariate adjustment as indicated in columns

*AA* ascending aorta; *AoD* aortic distensibility; *ASI* arterial stiffness index; *COVID-19* coronavirus disease 2019; *LVEDV* left ventricular endo-diastolic volume; *LVEF* left ventricular ejection fraction; *LVESV* left ventricular endo-systolic volume; *LVSV* left ventricular stroke volume; *GLS* global longitudinal strain; *MCS* circumferential strain at the mid short axis level; *MRS* radial strain at the mid short axis level; *PDA* proximal descending aorta; *RVEDV* right ventricular endo-diastolic volume; *RVEF* right ventricular ejection fraction; *RVESV* right ventricular end-systolic volume; *RVSV* right ventricular stroke volume

These relationships were unchanged when limiting analysis to the subset of participants tested in hospital (*n* = 244, positive *n* = 50) and, additionally, there was a significant association between smaller RV end-systolic volume and higher odds of COVID-19 positivity (Supplementary Table 5). In the sample with ASI measurement and COVID-19 testing (*n* = 6066), there was no association between ASI and death or critical care admission within the COVID-19 positive or negative cohorts in fully adjusted models (Supplementary Table 6).

## Discussion

### Summary of findings

In this sample of 310 UKB participants tested for COVID-19, we demonstrate association of pre-existing adverse morpho-functional CMR phenotypes with greater likelihood of COVID-19 positivity independent of classical cardiovascular risk factors. Specifically, in fully adjusted models, smaller LV and RV end-diastolic volume, lower LV stroke volume, and poorer GLS were associated with significantly higher odds of COVID-19 positive test. There were no significant associations between arterial stiffness (AoD, ASI) or native T1 and COVID-19 status.

### Comparison with existing research

Our study is the first to assess association of pre-existing cardiovascular phenotypes with subsequent COVID-19 outcomes.

In a cohort of 100 patients recovered from COVID-19, Puntmann et al. [23] report significantly larger LV end-diastolic volumes, lower LV/RV ejection fraction, smaller LV mass, higher native T1, higher native T2, and greater proportion of late gadolinium enhancement (LGE) abnormalities compared to risk factor matched controls. We observed similar significant associations between measures of poorer LV function (lower LV stroke volume, higher GLS) and COVID-19 positivity. However, we found no statistically significant association between native T1 and COVID-19 status in fully adjusted models. It is possible, that associations observed in our study indicate risk factors for symptomatic COVID-19, whilst the findings of Puntmann et al. [23] reflect alterations occurring after COVID-19. These different observations may also reflect different approaches to confounder adjustment. In our study, we consider a wider range of potential confounders, including, ethnicity, BMI, deprivation, and high cholesterol (in addition to all factors matched by Puntmann et al. [23]). Furthermore, our COVID-19 negative cohort comprise individuals tested for COVID-19, mostly in hospital (80.8%); therefore, these participants are likely to have been admitted with an acute illness, possibly with respiratory symptoms, during the same time period. The control subjects in Puntmann et al. [23] were not hospitalised or tested for COVID-19. Overall, our comparator cohort was more appropriately matched to the COVID-19 positive cases with consideration of a wider range of confounding variables. Therefore, some of the changes observed by Puntmann et al. [23], may be compromised by residual confounding. It is also possible that our study is underpowered to detect small associations with native T1.

Knight et al. [42] present a retrospective review of 29 individuals hospitalised with COVID-19, referred for CMR post-recovery with unexplained myocardial injury (elevated troponin) during the acute illness. Their participants had moderate-severe presentations of COVID-19, with over a third requiring intensive care and ventilatory support. There were non-ischaemic ("myocarditis-like") and ischaemic LGE patterns of myocardial injury in 13 and 2 participants, respectively. Interestingly, patients with "myocarditis-like" LGE did not have higher T2 (myocardial oedema) than the rest of the cohort, suggesting that either the observed myocardial alterations are fixed post-COVID-19 changes or that they pre-existed the infection. In a similar study, Huang et al. [25] report CMR findings from patients recovered from COVID-19 with ongoing cardiac symptoms. They present three-way and cross-group comparisons between patients with COVID-19 and positive CMR findings (defined as elevated T2 or presence of LGE, *n* = 15), COVID-19 and negative CMR findings (*n* = 11), and healthy controls (*n* = 20). They do not report any significant differences in LV volumetric or functional parameters. Compared to healthy controls, CMR positives had significantly poorer RV function by ejection fraction, stroke volume, and cardiac output. They report higher global native T1 in three way comparison, and when comparing CMR positives to healthy and CMR negative cases, but not when comparing CMR negatives with healthy controls. The CMR positive group had higher global T2 and extracellular volume (ECV) compared to healthy cohorts. As the researchers pre-select known abnormal cases (CMR positives) to compare with known normal cases (healthy comparators or CMR negatives), it is difficult to reliably compare these findings with those from our study.

Rajpal et al. [24] and Clark et al. [22] report CMR findings of young athletes (mean age 19.5 and 20.0 years, respectively) recovered from mild/asymptomatic COVID-19. Rajpal et al. [24] present a descriptive report of 26 college athletes, reporting non-ischaemic patterns of LGE in 12 participants, of whom, four also had elevated T2. Clark et al. [22] compare CMR metrics of 22 college athletes with 22 healthy controls and 22 tactical athletes. The COVID-19 positive group had significantly larger RV volumes and lower RV ejection fraction compared to both control groups. The results of parametric mapping indices showed no significant difference in native T1 between the groups, higher T2 in cases vs healthy controls, and higher ECV in cases vs tactical athletes, but not healthy controls. LGE was seen in two cases, the comparator groups did not have LGE imaging for comparison. It is possible that the observed changes related to athletic cardiac adaptation. Indeed, many of the volumetric and parametric mapping difference reported by Clark et al. [22] were less pronounced when comparing COVID-19 positive athletes to tactical athletic controls than healthy controls. Overall, interpretation of these non-specific CMR findings in these rather atypical populations is challenging, particularly as the participants had no symptoms or biochemical evidence of myocardial injury.

In a large multi-centre survey-based study, Dweck et al. [7] report echocardiographic findings from 1216 patients with acute COVID-19 and clinical indication for echocardiography performed in hospital. In patients without pre-existing cardiac disease, LV abnormalities were noted in 25% and RV abnormalities in 33%. Those with an abnormal scan were older and had higher prevalence of pre-existing disease. RV abnormalities were more common in patients with more severe COVID-19 and likely reflect elevations in RV afterload due to pulmonary embolism or pneumonia. LV abnormalities were predominantly non-specific, a few cases showed patterns consistent with myocardial infarction (3%), myocarditis (3%), or Takotsubo cardiomyopathy (2%). Mahmoud-Elsayed et al. [3] report similar findings in a single centre study of hospitalised patients with COVID-19 pneumonia. In this sample, as per Dweck et al. [7], RV abnormalities dominated, likely secondary to pulmonary pathology rather than cardiac involvement. Indeed, RV systolic dysfunction was significantly associated with elevated D-dimer and C reactive protein levels but not with high sensitivity Troponin I. In this cohort, LV systolic function was hyperdynamic or normal in most cases (89%); 11% had impaired LV systolic function and 4% had a dilated LV. These findings likely reflect changes in cardiac phenotypes occurring secondary to the acute COVID-19 illness, but also, perhaps cardiac features that predispose to symptomatic COVID-19; acute cardiac abnormalities that may have occurred due to COVID-19 appear to be uncommon and poorly defined with echocardiography.

## Clinical implications

Our findings demonstrate association of smaller LV and RV end-diastolic volume, lower LV stroke volume, and poorer GLS were with significantly higher odds of COVID-19 positive test. Overall, this pattern of associations presents the picture of a cardiac phenotype with poorer myocardial contractility and smaller stiffer ventricles. This suggests that adverse cardiac phenotypes, perhaps resembling a heart failure preserved ejection fraction (HFPEF) phenotype, are associated with greater odds of COVID-19. The magnitude of differences observed is small and, at the individual level, their clinical significance is highly uncertain. However, given the massive population burden of COVID-19, small effects in large number of people are likely to be important. Our findings of significant associations between pre-existing adverse CMR phenotypes and incident COVID-19 suggest that reports of long-term cardiac involvement of COVID-19 based on study of post-recovery CMR imaging may be hampered by residual confounding and reverse causation and that observed differences in CMR metrics in these studies may, at least partly, reflect pre-existing cardiac status rather than new COVID-19 related changes.

## Strengths and limitations

In the present study, we used a well-defined cohort, with CMR imaging performed according to uniform technical protocols, linked to national COVID-19 test results. Uniquely, imaging in this study preceded COVID-19 testing by 1–6 years. As a result, our findings add a new and important perspective to existing work, which exclusively relies on imaging performed after COVID-19 testing. The availability of data from a hospitalised COVID-19 negative cohort provided an appropriate comparator cohort. In the present study, case definition was based on SARS-CoV-2 RT-PCR. In practice, patients may be diagnosed with COVID-19 based on clinical presentation and investigations in the absence of a positive PCR test. However, misclassification of cases in our analysis would lead to a conservative bias if anything, and indeed in a sensitivity analysis including RT-PCR and clinically defined cases, our results were unchanged. Within the COVID-19 positive cohort, there were eight deaths and four critical care admissions, as such, we had very limited ability to discern whether baseline cardiac phenotypes were related to disease severity. Future analyses with greater numbers of events will be required to clarify this point. Whilst is it plausible that baseline cardiac phenotype might be associated with risk of symptoms that might occasion COVID-19 testing, it is unlikely that this would influence our findings since UK testing is limited to symptomatic SARS-CoV2 infection (indeed at the time of our analysis predominantly disease severe enough to require hospital admission), and we compared populations according to test result within the tested population. However, our results should be interpreted in the context of the more severe spectrum of COVID-19 manifestations and may not apply across milder or asymptomatic disease manifestations*.* Furthermore, due to the observational nature of the study, we cannot exclude residual confounding or infer causality.

## Conclusions

Our results, in a predominantly hospitalised cohort, demonstrate that several pre-existing adverse cardiac phenotypes are associated with greater risk of incident COVID-19, suggesting that these phenotypes may be risk factors for, rather than outcomes of, SARS-CoV-2 infection. Thus, observational reports of cardiovascular involvement after COVID-19 may, at least partly, reflect residual confounding and/or reverse causation from pre-existing cardiac status rather than COVID-19 induced alterations. However, whilst volumetric and ventricular function measures appeared dominant in our analysis, differences in tissue characteristics were more pronounced in studies reporting CMR phenotypes after COVID-19 infection. Thus, it is possible that whilst some adverse cardiac phenotypes pre-dispose to more severe COVID-19 and need for hospitalisation, SARS-CoV-2 infection itself might also lead to distinct phenotypic alterations. Further research in larger populations, with appropriate control groups and ideally imaging before and after COVID-19 disease, together with prospective follow-up, are required for definitive conclusions.

## Availability of data and material

Data used in this study are available to all bone fide researchers from UK Biobank through a formal access application: https://www.ukbiobank.ac.uk.

## Supplementary Information

Below is the link to the electronic supplementary material.

Supplementary file1 (DOCX 61 KB)

## Data Availability

Derived data will be returned to UK Biobank and made available to future researchers as per standard UK Biobank data return policy.
