# Supplementary material for: Adverse cardiovascular magnetic resonance phenotypes are associated with greater likelihood of incident coronavirus disease 2019: findings from the UK Biobank
Source: Aging Clin Exp Res. 2021 Mar 8;33(4):1133–44. doi: 10.1007/s40520-021-01808-z (PMC7938275; doi:10.1007/s40520-021-01808-z)
Supplement: Supplementary file 1 — Supplementary file1 (DOCX 61 KB) [file 40520_2021_1808_MOESM1_ESM.docx]

**Supplementary Table 1. International Classification of Disease (ICD) codes used to define comorbidities from Hospital Episode Statistic data**

| **Condition** | **ICD code** | **Code description** |
| --- | --- | --- |
| Diabetes | E100 | Type 1 diabetes mellitus: With coma |
| Diabetes | E101 | Type 1 diabetes mellitus: With ketoacidosis |
| Diabetes | E102 | Type 1 diabetes mellitus: With renal complications |
| Diabetes | E103 | Type 1 diabetes mellitus: With ophthalmic complications |
| Diabetes | E104 | Type 1 diabetes mellitus: With neurological complications |
| Diabetes | E105 | Type 1 diabetes mellitus: With peripheral circulatory complications |
| Diabetes | E106 | Type 1 diabetes mellitus: With other specified complications |
| Diabetes | E107 | Type 1 diabetes mellitus: With multiple complications |
| Diabetes | E108 | Type 1 diabetes mellitus: With unspecified complications |
| Diabetes | E109 | Type 1 diabetes mellitus: Without complications |
| Diabetes | E110 | Type 2 diabetes mellitus: With coma |
| Diabetes | E111 | Type 2 diabetes mellitus: With ketoacidosis |
| Diabetes | E112 | Type 2 diabetes mellitus: With renal complications |
| Diabetes | E113 | Type 2 diabetes mellitus: With ophthalmic complications |
| Diabetes | E114 | Type 2 diabetes mellitus: With neurological complications |
| Diabetes | E115 | Type 2 diabetes mellitus: With peripheral circulatory complications |
| Diabetes | E116 | Type 2 diabetes mellitus: With other specified complications |
| Diabetes | E117 | Type 2 diabetes mellitus: With multiple complications |
| Diabetes | E118 | Type 2 diabetes mellitus: With unspecified complications |
| Diabetes | E119 | Type 2 diabetes mellitus: Without complications |
| Diabetes | E130 | Other specified diabetes mellitus: With coma |
| Diabetes | E131 | Other specified diabetes mellitus: With ketoacidosis |
| Diabetes | E132 | Other specified diabetes mellitus: With renal complications |
| Diabetes | E133 | Other specified diabetes mellitus: With ophthalmic complications |
| Diabetes | E134 | Other specified diabetes mellitus: With neurological complications |
| Diabetes | E135 | Other specified diabetes mellitus: With peripheral circulatory complications |
| Diabetes | E136 | Other specified diabetes mellitus: With other specified complications |
| Diabetes | E137 | Other specified diabetes mellitus: With multiple complications |
| Diabetes | E138 | Other specified diabetes mellitus: With unspecified complications |
| Diabetes | E139 | Other specified diabetes mellitus: Without complications |
| Diabetes | E140 | Unspecified diabetes mellitus: With coma |
| Diabetes | E141 | Unspecified diabetes mellitus: With ketoacidosis |
| Diabetes | E142 | Unspecified diabetes mellitus: With renal complications |
| Diabetes | E143 | Unspecified diabetes mellitus: With ophthalmic complications |
| Diabetes | E144 | Unspecified diabetes mellitus: With neurological complications |
| Diabetes | E145 | Unspecified diabetes mellitus: With peripheral circulatory complications |
| Diabetes | E146 | Unspecified diabetes mellitus: With other specified complications |
| Diabetes | E147 | Unspecified diabetes mellitus: With multiple complications |
| Diabetes | E148 | Unspecified diabetes mellitus: With unspecified complications |
| Diabetes | E149 | Unspecified diabetes mellitus: Without complications |
| Diabetes | G590 | Diabetic mononeuropathy |
| Diabetes | G632 | Diabetic polyneuropathy |
| Diabetes | H280 | Diabetic cataract |
| Diabetes | H360 | Diabetic retinopathy |
| Diabetes | M142 | Diabetic arthropathy |
| Diabetes | N083 | Glomerular disorders in diabetes mellitus |
| Diabetes | O240 | Diabetes mellitus in pregnancy: Pre-existing type 1 diabetes mellitus |
| Diabetes | O241 | Diabetes mellitus in pregnancy: Pre-existing type 2 diabetes mellitus |
| Diabetes | O243 | Diabetes mellitus in pregnancy: Pre-existing diabetes mellitus, unspecified |
| Diabetes | O244 | Diabetes mellitus arising in pregnancy |
| Diabetes | O249 | Diabetes mellitus in pregnancy, unspecified |
| Diabetes | Y423 | Insulin and oral hypoglycaemic [antidiabetic] drugs |
| Hypertension | I10X | Essential (primary) hypertension |
| Hypertension | I110 | Hypertensive heart disease with (congestive) heart failure |
| Hypertension | I119 | Hypertensive heart disease without (congestive) heart failure |
| Hypertension | I120 | Hypertensive renal disease with renal failure |
| Hypertension | I129 | Hypertensive renal disease without renal failure |
| Hypertension | I130 | Hypertensive heart and renal disease with (congestive) heart failure |
| Hypertension | I131 | Hypertensive heart and renal disease with renal failure |
| Hypertension | I132 | Hypertensive heart and renal disease with both (congestive) heart failure and renal failure |
| Hypertension | I139 | Hypertensive heart and renal disease, unspecified |
| High cholesterol | E780 | Pure hypercholesterolaemia |
| High cholesterol | E782 | Mixed hyperlipidaemia |
| High cholesterol | E783 | Hyperchylomicronaemia |
| High cholesterol | E784 | Other hyperlipidaemia |
| High cholesterol | E785 | Hyperlipidaemia, unspecified |

**Supplementary Table 2. Baseline characteristics by mortality and critical care outcomes in COVID-19 positives and negatives**

|  | **COVID-19 positive (n= 70)** | | | | **COVID-19 negative (n= 240)** | | | |
| --- | --- | --- | --- | --- | --- | --- | --- | --- |
|  | **Alive**  **n=62** | **Dead n=8** | **No critical care n=66** | **Critical care n=4** | **Alive n=229** | **Dead n=11** | **No critical care n=233** | **Critical care n=7** |
| **Age** | 60.4 [55.0, 67. 1] | 78.0 [75.7, 81.0] | 62.1 [55.5, 72.4] | 59.6 [57.6, 64.0] | 64.0 [58.0, 72.0] | 71.2 [66.2, 75.1] | 65.1 [58.1, 72.1] | 66.5 [61.0, 71.8] |
| **Sex (Male)** | 35 (56.5%) | 6 (75.0%) | 37 (56.1%) | 4 (100.0%) | 111 (48.5%) | 6 (54.5%) | 111 (47.6%) | 6 (85.7%) |
| **White** | 55 (88.7%) | 8 (100.0%) | 59 (89.4%) | 4 (100.0%) | 221 (96.5%) | 11 (100.0%) | 225 (96.6%) | 7 (100.0%) |
| **BAME** | 7 (11.3%) |  | 7 (10.6%) |  | 8 (3.5%) |  | 8 (3.4%) |  |
| **Asian** | 3 (4.8%) |  | 3 (4.5%) |  | 5 (2.2%) |  | 5 (2.1%) |  |
| **Black** | 2 (3.2%) |  | 2 (3.0%) |  |  |  |  |  |
| **Mixed** | 1 (1.6%) |  | 1 (1.5%) |  | 2 (0.9%) |  | 2 (0.9%) |  |
| **Other** | 1 (1.6%) |  | 1 (1.5%) |  | 1 (0.4%) |  | 1 (0.4%) |  |
| **Townsend score** | -2.2 [-4.0, 0.4] | -3.6 [-4.2, -0.8] | -2.4 [-4.2, 0.3] | 0.2 [-3.3, 3. 8] | -2.6 [-3.9, 0.2] | -2.4 [-3. 7, -1.2] | -2.5 [-3.8, 0.0] | -2.6 [-3.1, 0.7] |
| **Smoking*** | 27 (43.5%) | 5 (62.5%) | 30 (45.5%) | 2 (50.0%) | 102 (44.5%) | 6 (54.5%) | 105 (45.1%) | 3 (42.9%) |
| **BMI** | 27.4 (± 5.9) | 29.3 (± 1. 9) | 27.3 (± 5. 6) | 32.9 (± 4.9) | 27.3 (± 4.5) | 27.5 (± 7.4) | 27.2 (± 4.7) | 28.5 (± 5. 0) |
| **Diabetes** | 4 (6.5%) | 1 (12.5%) | 4 (6.1%) | 1 (25.0%) | 23 (10.0%) | 1 (9.1%) | 23 (9.9%) | 1 (14.3%) |
| **Hypertension** | 20 (32.3%) | 5 (62.5%) | 23 (34.8%) | 2 (50.0%) | 93 (40.6%) | 8 (72.7%) | 96 (41.2%) | 5 (71.4%) |
| **High cholesterol** | 17 (27.4%) | 4 (50.0%) | 18 (27.3%) | 3 (75.0%) | 64 (27.9%) | 6 (54.5%) | 69 (29.6%) | 1 (14.3%) |
| **Prior AMI** | 1 (1.6%) |  | 1 (1.5%) |  | 7 (3.1%) | 3 (27.3%) | 10 (4.3%) |  |
| **Tested in hospital** | 43 (69.4%) | 7 (87.5%) | 47 (71.2%) | 3 (75.0%) | 185 (80.8%) | 9 (81.8%) | 189 (81.1%) | 5 (71.4%) |

**Supplementary Table 2 caption.** Green shading indicates p-value of difference between 0.1–0.05. Yellow shading indicated p-value of difference <0.05. *smoking indicated previous/current smoking. AMI: acute myocardial infarction; BAME: Black, Asian, and minority ethnic; BMI: body mass index; COVID-19: coronavirus disease 2019

**Supplementary Table 3. Baseline population characteristics (ASI set)**

|  | **Whole sample**  **n=6,066** | **COVID negatives**  **n=5,399** | **COVID positives**  **n=667** | **p-value [test]** |
| --- | --- | --- | --- | --- |
| **Age** | 70.9 [62.2, 75.7] | 71.0 [62.8, 75.8] | 68.1 [59.0, 75.1] | 4.4x10^-7^ [2] |
| **Sex (Male)** | 2,947 (48.6%) | 2,586 (47.9%) | 361 (54.1%) | 2.8x10^-3^ [3] |
| **White** | 5,357 (88.3%) | 4,810 (89.1%) | 547 (82.0%) | 1.5x10^-7^ [4] |
| **BAME** | 660 (10.9%) | 545 (10.1%) | 115 (17.2%) |  |
| **Asian** | 288 (4.7%) | 243 (4.5%) | 45 (6.7%) | 5.0x10^-4^ [4] |
| **Black** | 208 (3.4%) | 162 (3.0%) | 46 (6.9%) |  |
| **Chinese** | 15 (0.2%) | 12 (0.2%) | 3 (0.4%) |  |
| **Mixed** | 42 (0.7%) | 38 (0.7%) | 4 (0.6%) |  |
| **Other** | 156 (2.6%) | 134 (2.5%) | 22 (3.3%) |  |
| **Townsend deprivation score** | -1.4 [-3.2, 1.4] | -1.5 [-3.2, 1.4] | -0.8 [-3.0, 2.1] | 1.0x10^-3^ [2] |
| **Smoking (current/previous)** | 2,946 (48.6%) | 2,609 (48.3%) | 337 (50.5%) | 0.302 [3] |
| **BMI** | 28.3 (± 5.1) | 28.2 (± 5.1) | 28.7 (± 5.12) | 0.023 [1] |
| **Diabetes** | 954 (15.7%) | 817 (15.1%) | 137 (20.5%) | 3.7x10^-4^ [3] |
| **Hypertension** | 3,073 (50.7%) | 2,731 (50.6%) | 342 (51.3%) | 0.767 [3] |
| **High cholesterol** | 2,253 (37.1%) | 2,017 (37.4%) | 236 (35.4%) | 0.340 [3] |
| **Prior myocardial infarction** | 397 (6.5%) | 348 (6.4%) | 49 (7.3%) | 0.421 [3] |
| **ASI (m/s)** | 9.3 (± 2.9) | 9.3 (± 2.9) | 9.2 (± 2.7) | 0.348 [1] |
| **Testing in hospital** | 4,546 (74.9%) | 4,128 (76.5%) | 418 (62.7%) | 1.3x10^-14^ [3] |
| **Critical care admission** | 149 (2.5%) | 97 (1.8%) | 52 (7.8%) | 1.3x10^-20^ [3] |
| **Death** | 347 (5.7%) | 217 (4.0%) | 130 (19.5%) | 1.3x10^-58^ [3] |

**Supplementary Table 3 caption.** [1] Welch Two Sample t-test (numeric data with unequal variances); [2] Wilcoxon rank sum test with continuity correction (numeric skewed); [3] Two-sample test for equality of proportions with continuity correction (where minimum count > 5); [4] Fisher's Exact Test for Count Data (where minimum count $\leq$5). BAME: Black, Asian, and minority ethnic; BMI: body mass index; COVID-19: coronavirus disease 2019.

**Supplementary Table 4. Cardiovascular phenotypes by mortality and critical care outcome in COVID-19 positives and negatives**

|  | **COVID-19 positive (n= 70)** | | | | **COVID-19 negative (n= 240)** | | | |
| --- | --- | --- | --- | --- | --- | --- | --- | --- |
|  | **Alive**  **n=62** | **Dead n=8** | **No critical care n=66** | **Critical care n=4** | **Alive n=229** | **Dead n=11** | **No critical care n=233** | **Critical care n=7** |
| **LVEDVi (ml/m^2^)** | 77.5 (± 13.2) | 73.2 (± 13.3) | 76.8 (± 13.4) | 81.5 (± 8.1) | 81.0 (± 14.4) | 76.2 (± 14.1) | 80.6 (± 13.8) | 89.4 (± 26.4) |
| **LVESVi (ml/m^2^)** | 31.1 (± 7.8) | 33.9 (± 10.4) | 31.3 (± 8.2) | 32.3 (± 6.0) | 31.6 (± 8.2) | 31.0 (± 12.4) | 31.5 (± 8.2) | 33.3 (± 14.0) |
| **LVSVi (ml/m^2^)** | 46.5 (± 8.3) | 39.3 (± 6.5) | 45.5 (± 8.4) | 49.3 (± 9.3) | 49.4 (± 9.5) | 45.2 (± 7.7) | 49.0 (± 9.2) | 56.1 (± 15.9) |
| **LVEF (%)** | 60.2 (± 6.2) | 54.3 (± 7.1) | 59.5 (± 6.5) | 60.2 (± 7.7) | 61.2 (± 6.2) | 60.4 (± 10.1) | 61.1 (± 6.4) | 63.0 (± 9.1) |
| **LVMi (g/m^2^)** | 45.9 (± 8.4) | 44.5 (± 9.2) | 45.3 (± 8.4) | 53.3 (± 5.9) | 46.8 (± 8.6) | 50.2 (± 13.3) | 46.6 (± 8.7) | 55.9 (± 10.3) |
| **RVEDVi (ml/m^2^)** | 77.5 (± 15.0) | 71.5 (± 15.2) | 76.9 (± 15.2) | 76.7 (± 13.4) | 80.1 (± 15.7) | 74.3 (± 12.2) | 79.8 (± 15.3) | 83.2 (± 24.8) |
| **RVESVi (ml/m^2^)** | 30.1 (± 8.0) | 29.3 (± 9.3) | 29.9 (± 8.2) | 32.5 (± 6.0) | 31.1 (± 8.5) | 28.5 (± 8.9) | 30.8 (± 8.2) | 36.0 (± 15.8) |
| **RVSVi (ml/m^2^)** | 47.4 (± 10.0) | 42.2 (± 7.1) | 47.0 (± 10.0) | 44.1 (± 7.4) | 49.0 (± 10.6) | 45.7 (± 8.6) | 48.9 (± 10.5) | 47.2 (± 11.5) |
| **RVEF (%)** | 61.3 (± 6.3) | 59.5 (± 5.1) | 61.3 (± 6.4) | 57.6 (± 0.6) | 61.3 (± 6.7) | 61.9 (± 9.0) | 61.4 (± 6.7) | 57.5 (± 6.5) |
| **T1 (ms)** | 924.8 (± 37.0) | 919.0 (± 26.4) | 924.3 (± 36.7) | 921.0 (± 10.7) | 922.7 (± 39.3) | 928.3 (± 54.4) | 923.5 (± 40.2) | 904.8 (± 31.7) |
| **MRS (%)** | 35.4 (± 8.4) | 30.2 (± 7.7) | 34.9 (± 8.7) | 34.6 (± 4.3) | 35.6 (± 9.0) | 36.6 (± 14.6) | 35.8 (± 9.4) | 31.0 (± 4.7) |
| **MCS (%)** | -20.0 (± 2.9) | -17.8 (± 3.9) | -19.8 (± 3.1) | -20.0 (± 1.5) | -20.1 (± 3.1) | -20.0 (± 4.3) | -20.1 (± 3.2) | -18.6 (± 1.8) |
| **GLS (%)** | -15.4 (± 2.4) | -11.8 (± 1.8) | -15.0 (± 2.7) | -15.0 (± 2.1) | -15.6 (± 2.5) | -14.2 (± 2.2) | -15.5 (± 2.6) | -15.4 (± 1.5) |
| **Torsion (degrees)** | 0.8 (± 0.9) | 0.5 (± 1.7) | 0.8 (± 1.0) | 0.7 (± 1.0) | 0.9 (± 0.8) | 1.0 (± 0.3) | 0.9 (± 0.7) | 1.1 (± 0.5) |
| **AA AoD (× 10^-3^ mmHg^-1^)** | 1.5 [1.0, 2.6] | 0.7 [0.4, 1.3] | 1.4 [0.9, 2.3] | 1.6 [1.1, 2.6] | 1.4 [0.8, 2.4] | 1.0 [0.6, 1.3] | 1.3 [0.8, 2.3] | 2.5 [1.3, 3.7] |
| **PDA AoD (× 10^-3^ mmHg^-1^)** | 2.4 [1.8, 3.5] | 1.6 [0.8, 2.1] | 2.4 [1.6, 3.3] | 1.8 [1.8, 2.3] | 2.2 [1.7, 3.1] | 2.1 [1.4, 2.4] | 2.2 [1.7, 3.1] | 2.2 [1.8, 4.3] |
| **ASI (m/s)** | 9.1 (± 2.7) | 9.7 (± 2.7) | 9.2 (± 2.8) | 9.6 (± 2.2) | 9.3 (± 2.9) | 9.8 (± 2.8) | 9.3 (± 2.9) | 10.0 (± 2.8) |

**Supplementary Table 4 caption:** Green shading indicates p-value of difference between 0.1–0.05. Yellow shading indicated p-value of difference <0.05 AA: ascending aorta; AoD: aortic distensibility; COVID-19: coronavirus disease 2019; LVEDV: left ventricular endo-diastolic volume; LVEF: left ventricular ejection fraction; LVESV: left ventricular endo-systolic volume; LVSV: left ventricular stroke volume; GLS: global longitudinal strain; MCS: circumferential strain at the mid short axis level; MRS: radial strain at the mid short axis level; PDA: proximal descending aorta; RVEDV: right ventricular endo-diastolic volume; RVEF: right ventricular ejection fraction; RVESV: right ventricular end-systolic volume; RVSV: right ventricular stroke volume- shading indicates comparison between dead vs alive and critical care vs no critical care within the test positive and test negative cohorts.

**Supplementary Table 5. Odds ratios from logistic regression models demonstrating association of cardiovascular phenotype measures with COVID-19 status in the subset tested in hospital**

|  | **Univariate** | **Age and Sex Adjusted** | **Fully Adjusted** |
| --- | --- | --- | --- |
| **LVEDVi (ml/m2)** | 0.98 [0.96, 1.00] | 0.97* [0.94, 0.99] | 0.97* [0.94, 1.00] |
|  | 0.064 | 0.010 | 0.028 |
| **LVESVi (ml/m2)** | 1.00 [0.96, 1.04] | 0.98 [0.94, 1.02] | 0.99 [0.95, 1.04] |
|  | 0.899 | 0.338 | 0.755 |
| **LVSVi (ml/m2)** | 0.95* [0.92, 0.99] | 0.94* [0.91, 0.98] | 0.94* [0.90, 0.98] |
|  | 7.2$\times$10^-3^ | 2.5$\times$10^-3^ | 3.9$\times$10^-3^ |
| **LVEF (%)** | 0.96 [0.91, 1.01] | 0.97 [0.92, 1.02] | 0.95 [0.90, 1.01] |
|  | 0.078 | 0.194 | 0.089 |
| **LVMi (g/m2)** | 0.99 [0.95, 1.02] | 0.95* [0.91, 1.00] | 0.96 [0.92, 1.01] |
|  | 0.459 | 0.047 | 0.137 |
| **RVEDVi (ml/m2)** | 0.98 [0.96, 1.00] | 0.97* [0.95, 0.99] | 0.97* [0.95, 1.00] |
|  | 0.098 | 0.014 | 0.022 |
| **RVESVi (ml/m2)** | 0.98 [0.94, 1.01] | 0.95* [0.90, 0.99] | 0.95* [0.91, 1.00] |
|  | 0.244 | 0.018 | 0.049 |
| **RVSVi (ml/m2)** | 0.98 [0.95, 1.01] | 0.97 [0.94, 1.00] | 0.97 [0.93, 1.00] |
|  | 0.125 | 0.070 | 0.065 |
| **RVEF (%)** | 1.00 [0.96, 1.05] | 1.03 [0.97, 1.08] | 1.02 [0.96, 1.07] |
|  | 0.911 | 0.341 | 0.581 |
| **T1 (ms)** | 1.00 [0.99, 1.01] | 1.01 [1.00, 1.01] | 1.00 [1.00, 1.01] |
|  | 0.645 | 0.258 | 0.287 |
| **MRS (%)** | 0.98 [0.95, 1.02] | 1.00 [0.96, 1.04] | 0.99 [0.94, 1.03] |
|  | 0.303 | 0.882 | 0.567 |
| **MCS (%)** | 1.05 [0.95, 1.16] | 1.01 [0.91, 1.13] | 1.04 [0.93, 1.17] |
|  | 0.294 | 0.802 | 0.478 |
| **GLS (%)** | 1.12 [0.99, 1.26] | 1.14 [1.00, 1.31] | 1.18* [1.02, 1.37] |
|  | 0.072 | 0.053 | 0.026 |
| **Torsion (degrees)** | 0.86 [0.60, 1.25] | 0.88 [0.60, 1.31] | 0.88 [0.60, 1.31] |
|  | 0.418 | 0.509 | 0.527 |
| **AA AoD (× 10^-3^ mmHg^-1^)** | 1.12 [0.84, 1.48] | 0.99 [0.67, 1.38] | 1.00 [0.67, 1.42] |
|  | 0.425 | 0.938 | 0.983 |
| **PDA AoD (× 10^-3^ mmHg^-1^)** | 1.12 [0.85, 1.47] | 1.04 [0.74, 1.42] | 1.09 [0.76, 1.52] |
|  | 0.409 | 0.832 | 0.637 |
| **ASI (m/s)** | 0.99 [0.96, 1.02] | 0.98 [0.95, 1.01] | 0.98 [0.95, 1.02] |
|  | 0.528 | 0.348 | 0.391 |

**Supplementary Table 5 caption.** Analysis sample n=244 (n= 50 positive); Modelling is with sample tested for COVID-19 with analysable CMR data. Model outcome is set as COVID-19 test result (positive vs negative). Fully adjusted model includes adjustment for age, sex, ethnicity, deprivation, body mass index, smoking, diabetes, hypertension, high cholesterol, and prior myocardial infarction. Results are odds ratio [95% confidence interval] and p-value, each belonging to a separate logistic regression model with covariate adjustment as indicated in columns. Abbreviations: AA: ascending aorta; AoD: aortic distensibility; ASI: arterial stiffness index; COVID-19: coronavirus disease 2019; LVEDV: left ventricular endo-diastolic volume; LVEF: left ventricular ejection fraction; LVESV: left ventricular endo-systolic volume; LVSV: left ventricular stroke volume; GLS: global longitudinal strain; MCS: circumferential strain at the mid short axis level; MRS: radial strain at the mid short axis level; PDA: proximal descending aorta; RVEDV: right ventricular endo-diastolic volume; RVEF: right ventricular ejection fraction; RVESV: right ventricular end-systolic volume; RVSV: right ventricular stroke volume.

**Supplementary Table 6. Logistic regression models demonstrating association of arterial stiffness index with COVID-19 status, death, and critical care admission in different sample subsets**

| **Sample** | **Outcome** | **Univariate** | **Age and Sex Adjusted** | **Fully Adjusted** |
| --- | --- | --- | --- | --- |
| Whole sample | COVID-19 test result | 0.99 [0.96, 1.02] | 0.99 [0.96, 1.02] | 0.99 [0.96, 1.02] |
|  |  | 0.367 | 0.549 | 0.481 |
| Tested in hospital | COVID-19 test result | 0.99 [0.95, 1.02] | 0.98 [0.95, 1.02] | 0.98 [0.95, 1.02] |
|  |  | 0.528 | 0.348 | 0.391 |
| COVID-19 negatives | Death | 1.06* [1.01, 1.11] | 1.02 [0.97, 1.07] | 1.01 [0.97, 1.07] |
|  |  | 0.017 | 0.486 | 0.553 |
| COVID-19 positives | Death | 1.08* [1.01, 1.16] | 1.00 [0.92, 1.07] | 0.98 [0.91, 1.06] |
|  |  | 0.032 | 0.899 | 0.699 |
| COVID-19 negatives | Critical care admission | 1.08* [1.01, 1.16] | 1.07 [1.00, 1.15] | 1.07 [0.99, 1.15] |
|  |  | 0.021 | 0.064 | 0.072 |
| COVID-19 positives | Critical care admission | 1.06 [0.96, 1.17] | 1.03 [0.92, 1.15] | 1.02 [0.91, 1.14] |
|  |  | 0.268 | 0.601 | 0.758 |

**Supplementary Table 6 caption:** Fully adjusted model includes adjustment for age, sex, ethnicity, deprivation, body mass index, smoking, diabetes, hypertension, high cholesterol, and prior myocardial infarction. Results are odds ratio [95% confidence interval] and p-value, each belonging to a separate logistic regression model with covariate adjustment as indicated in columns. Abbreviations: COVID-19: coronavirus disease 2019.
